# Symptomatic Hypercalcemia in Patients with Primary Hyperparathyroidism Is Associated with Severity of Disease, Polypharmacy, and Comorbidity

**DOI:** 10.1155/2019/7617254

**Published:** 2019-12-30

**Authors:** C. Aresta, E. Passeri, S. Corbetta

**Affiliations:** ^1^Endocrine Unit, IRCCS Istituto Auxologico Italiano, Milan, Italy; ^2^Endocrinology and Diabetology Service, IRCCS Istituto Ortopedico Galeazzi, Milan, Italy; ^3^Department of Biomedical, Surgical and Dental Sciences, University of Milan, Milan, Italy

## Abstract

Current primary hyperparathyroidism (PHPT) clinical presentation is asymptomatic in more than 90% of patients, while symptoms concern osteoporosis and rarely kidney stones. Here, we retrospectively investigated the prevalence of PHPT patients presenting with hypercalcemic-related symptoms (HS-PHPT) as cognitive impairment, changes in sensorium, proximal muscle weakness, nausea and vomiting, constipation, and severe dehydration, in a single center equipped with an emergency department and described their clinical features and outcome in comparison with a series of asymptomatic PHPT out-patients (A-PHPT). From 2006 to 2016, 112 PHPT patients were consecutively diagnosed: 16% (*n* = 18, 3M/15F) presented with hypercalcemic-related symptoms. Gastrointestinal symptoms occurred in 66% of HS-PHPT patients and cognitive impairment in 44%; one woman experienced hypertensive heart failure. Two-thirds of HS-PHPT patients were hospitalized due to the severity of symptoms. Comparing the clinical features of HS-PHPT patients with A-PHPT patients, no gender differences were detected in the two groups, while HS-PHPT patients were older at diagnosis (71 (61–81) vs. 64 (56–74) years, *P*=0.04; median (IQR)). HS-PHPT patients presented higher albumin-corrected calcium levels (12.3 (11.3–13.7) vs. 10.6 (10.3–11.3) mg/dl, *P* < 0.001); 4 HS-PHPT presented corrected calcium levels >14 mg/dl. Serum PTH levels and total alkaline phosphatase activity were higher in HS-PHPT. Reduced kidney function (eGFR < 45 ml/min) was prevalent in HS-PHPT patients (42% vs. 5%, *P*=0.05). No differences in kidney stones and osteoporosis were detected, as well as in the rates of cardiovascular comorbidities and main cardiovascular risk factors. HS-PHPT patients had an age-adjusted Charlson Comorbidity Index higher than that of the A-PHPT patients and were on chronic therapy with a greater number of medications than A-PHPT patients. In conclusion, hypercalcemic-related symptoms occurred in 16% of PHPT patients. Risk factors were severity of the parathyroid tumor function, multimorbidity, and polypharmacy.

## 1. Introduction

Primary hyperparathyroidism (PHPT) is the third most common endocrine disorder, after diabetes and thyroid diseases, characterized by an inappropriate secretion of parathyroid hormone (PTH) from parathyroid glands. The incidence of PHPT significantly increased in the last decades in western countries, probably due to the inclusion of serum calcium level determination in biochemical screening tests for osteoporosis evaluation [1]. As a result, even the PHPT clinical presentation has changed from symptomatic disorder, primarily characterized by overt skeletal fragility, kidney stones, and hypercalcemic symptoms, to asymptomatic one, that accounts for more than 90% of cases in USA. Due to poor clinical symptoms, PHPT is often an incidental diagnosis during routine evaluation for osteoporosis in postmenopausal women.

PHPT is the most common cause of hypercalcemia [2], although a normocalcemic variant has been established. Hypercalcemia may be associated with a spectrum of clinical manifestations that usually correlate with the absolute level of serum calcium and the rapidity of onset. Mild calcium levels elevation is often well tolerated, with, when present, aspecific symptoms as fatigue or constipation. More severe clinical presentation, including polyuria, dehydration, anorexia, nausea, and changes in sensorium can be associated with a severe and/or acute rise in serum calcium levels, and patients with severe clinical conditions may require acute hospitalization for the management. However, most PHPT patients are nowadays managed as out-patients.

The aims of the study were (1) to determine the prevalence of PHPT patients presenting with symptomatic hypercalcemia (HS-PHPT) at a single center equipped with an emergency department; (2) to describe their clinical and biochemical features and outcomes in comparison with a series of asymptomatic PHPT (A-PHPT) out-patients.

## 2. Patients and Methods

### 2.1. Patients

We retrospectively analyzed data from 112 consecutive patients with PHPT (21 males and 91 females) referred to the single academic center (IRCCS Policlinico San Donato, San Donato Milanese, Milan, Italy) from 2006 to 2016. All patients were white Caucasians.

PHPT was diagnosed when hypercalcemia, namely elevated albumin-corrected serum calcium and/or ionized calcium, and elevated or inappropriately normal PTH level occurred. Patients with normocalcemic PHPT, characterized by elevated PTH levels and normal serum calcium levels, in the absence of any other causes for secondary elevation of PTH, such as severe vitamin D deficiency or renal failure [3], were included.

Symptomatic hypercalcemia was defined as occurrence of hypercalcemic-related symptoms, namely cognitive impairment, severe nausea and vomiting, proximal muscle hypostenia, and hemodynamic instability in patients with diagnosis of PHPT.

The study was conducted in accordance with the Declaration of Helsinki. This study was carried out in accordance with the recommendations of IRCCS Ospedale San Raffaele Milan Ethical Committee with written informed consent from all subjects. All subjects gave written informed consent in accordance with the Declaration of Helsinki. The protocol was approved by the local Ethical Committee.

### 2.2. Methods

Data were retrospectively collected from the medical record including details on hospital admission, personal and familial medical history, anthropometric measurements, including height and weight, and detailed medication lists. We distinguished hypercalcemia-related symptoms in four main categories: gastrointestinal manifestations (anorexia, nausea, vomiting, abdominal pain, and constipation), neurologic symptoms (cognitive impairment, irritability, and changes in sensorium), musculoskeletal manifestations (fatigue and proximal muscle weakness), and hemodynamic instability (dehydration and hypotension).

Weight was measured on a standard balance beam scale; height was measured using a calibrated, wall-mounted Harpenden stadiometer. Body mass index (BMI) was calculated.

Age-adjusted Charlson Comorbidity Index (ACCI) was determined for each patient by taking into account the occurrence of specific pathologic conditions, with additional points added for age (https://www.mdcalc.com/charlson-comorbidity-index-cci) [4].

Overnight fasting biochemical evaluation included serum and 24-hour urinary calcium, serum albumin, ionized calcium, serum and 24-hour urinary phosphate, total alkaline phosphatase, bone alkaline phosphatase isoenzymes, glucose, insulin, total and HDL cholesterol, triglycerides, and creatinine. Biochemical parameters were assayed by routine methods (Roche modular platform, Roche Diagnostics, Mannheim, Germany); in particular, albumin was measured immunoturbidimetrically, providing measurements similar to those obtained by the bromocresol purple (BCP)-based method [5]. The albumin-corrected serum calcium was calculated according to the following formula: serum total calcium (mg/dL) − 0.8 × (serum albumin (g/dL) − 4.0). Ionized calcium concentrations were assayed by Liquichek Blood Gas (IL Synthesis Series, BioRad Laboratories, Segrate, MI, Italy). Hormonal parameters, namely serum intact PTH and 25-hydroxyvitamin D (25OHD) were assayed by Electrochemiluminescence on an Elecsys 2010 (Roche Diagnostics, Mannheim, Germany) (mean intra-assay and interassay coefficients of variation (CV) of 2.3% and 3%, respectively) and LIAISON® 25OHvitamin D total assay (DiaSorin Inc., Stillwater, MN, USA) with mean intra- and interassay CVs of 4.5% and 7.5%, respectively. The estimated glomerular filtration rate (eGFR) was calculated by the CKD-EPI creatinine equation [6]. Insulin resistance was evaluated according to the HOMA-IR formula [7].

All patients were evaluated with a dual-energy X-ray absorptiometry (DXA) to measure BMD at the lumbar spine (LS), total femur (FT), and femoral neck (FN). Osteoporosis was diagnosed by a BMD T-score lower than −2.5 in postmenopausal patients and by a BMD Z-score lower than −2.0 in premenopausal patients according to the World Health Organization criteria [8]. Ultrasound kidney examination was obtained in all patients.

### 2.3. Statistical Analysis

The normality of distribution was tested by the Kolmogorov–Smirnov test. Data were nonparametric and were presented as median and interquartile (IQ) range for continuous parameters. Categorical data were presented as percentages. Differences between nonnormally distributed parameters were investigated by the Mann–Whitney *U* test. Differences between frequencies were analysed by the Fischer exact test. The receiver operating characteristic (ROC) curve analysis was performed to assess for each parameter found to be statistically different between HS-PHPT and A-PHPT patients and the predictivity of the occurrence of the hypercalcemia-related symptoms. The logistic regression analysis assessed the association between polypharmacy and age-adjusted Charlson index and the risk to develop hypercalcemic-related symptoms, after adjustment for age and eGFR. A *P* value less than 0.05 was considered significant. Statistical analysis was performed using Prism 6.0.

## 3. Results

### 3.1. Prevalence of Symptomatic Hypercalcemia in the Series of PHPT Patients

Eighteen patients experienced symptomatic hypercalcemia out of a total of 112 newly diagnosed PHPT patients, indicating a prevalence of 16%. The clinical severity of the SH required hospitalization in the two thirds of cases (*n* = 12).

The most frequent manifestations were gastrointestinal symptoms, occurring in 66% of hypercalcemic-related symptomatic patients (HS-PHPT), the leading ones being nausea and anorexia. Neurologic symptoms, most commonly mild cognitive impairment and irritability, occurred in 50% of cases. Musculoskeletal manifestations were present in 5 patients (27%), and hemodynamic instability, namely lipothymic episodes, was reported in 5 patients (27%). One woman presented hypertensive congestive heart failure, while sepsis occurred in one 50-year-old woman.

### 3.2. Managing HS-PHPT Patients

Parathyroidectomy was performed in 10 out of 18 (55%) HS-PHPT patients: 9 patients were diagnosed with parathyroid adenoma, and one patient affected with chronic renal failure presented diffuse parathyroid hyperplasia. One patient died 20 days after parathyroidectomy due to major hemorrhagic stroke. Transient hypoparathyroidism, resolving within six months, occurred in 8 patients, whereas only one patient resulted with permanent hypoparathyroidism. The patient with parathyroid hyperplasia experienced a reduction in calcium levels, without complete normalization.

Five HS-PHPT patients were not suitable to parathyroidectomy due to high surgical risk and were successfully treated with oral cinacalcet HCl. Two patients presented mildly elevated calcium levels (less than 11.0 mg/dl) after the resolution of the precipitating event and dehydration. One patient was lost at the follow-up.

### 3.3. Comparison between HS-PHPT and A-PHPT Patients

Data are presented in Table 1. HS-PHPT patients were significantly older than A-PHPT patients, while no gender differences were detected in the two groups. Multiple endocrine neoplasia type 1 (MEN1) syndrome was diagnosed in 8 (7%) PHPT patients, though none of the MEN1-related PHPT patients experienced SH.

**Figure 1:**
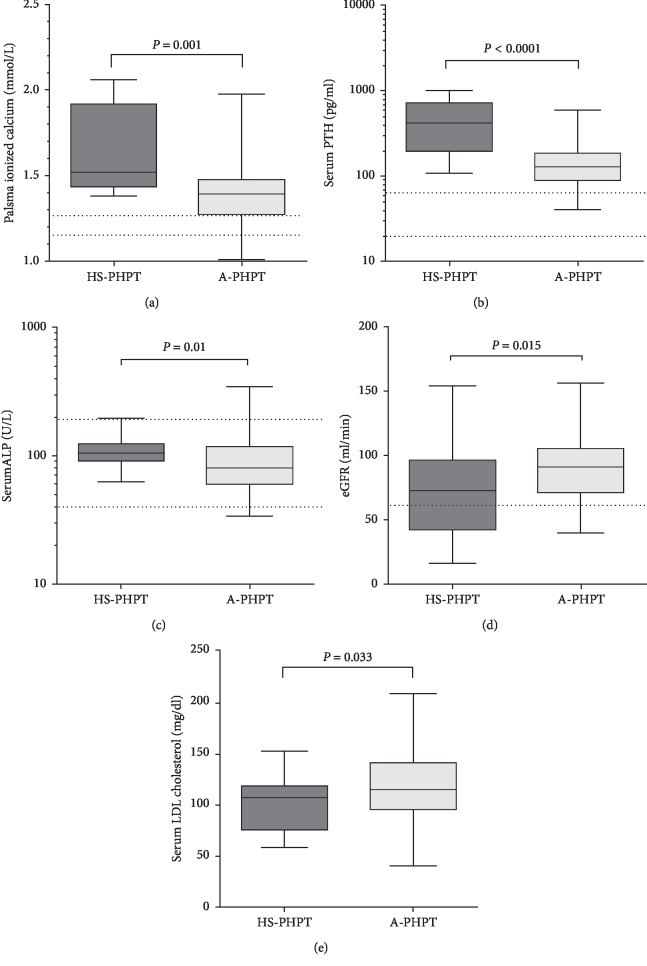
Differences in biochemical parameters between HS-PHPT and A-PHPT. Median plasma ionized calcium (a), serum PTH (b), serum total ALP activity (c), eGFR (d), and LDL cholesterol (e) significantly differed between HS-PHPT and A-PHPT patients. Dashed lines indicate normal reference ranges. Data are presented as median and range interquartile by box, and wisker plots represent minimum and maximum values. Statistical significance was determined by the Mann–Whitney test. HS-PHPT, hypercalcemic-related symptomatic PHPT (dark grey boxes); A-PHPT, asymptomatic PHPT (light grey boxes); PTH, parathormone; ALP, alkaline phosphatase; eGFR, estimated glomerular filtration rate; and LDL, low density lipoprotein.

HS-PHPT patients presented significantly higher median albumin-corrected calcium levels than A-PHPT patients, and 4 patients had albumin-corrected calcium levels above 14.0 mg/dl. Moreover, median serum ionized calcium, PTH levels, and total alkaline phosphatase (ALP) were higher in HS-PHPT patients (Figures 1(a)–1(c), panels). Estimated glomerular filtration rate (eGFR) was significantly lower in HS-PHPT (Figure 1(d), panel), and reduced kidney function (eGFR < 45 ml/min; CKD stage G3b and severer) was about 8 folds more frequently detected in HS-PHPT patients. Median serum 25OHD levels were similar in the two groups of PHPT patients, as well as median serum phosphate and 24-hours urinary calcium levels, while median 24-hours urine phosphate levels were lower in HS-PHPT vs. A-PHPT patients, likely related to the lower eGFR.

Received operator characteristic curve (ROC curve) analysis showed that serum calcium levels were significantly associated with hypercalcemic-related symptoms in PHPT patients (area 0.866, *P* < 0.0001), and the threshold of 12.0 mg/dl predicted the occurrence of hypercalcemic-related symptoms with a sensitivity of 93.6% and a specificity of 61.1%. Moreover, serum PTH and ALP levels were associated with hypercalcemic-related symptoms (area 0.880 and 0.702, *P* < 0.0001 and *P*=0.010, respectively).

Considering PHPT clinical features, the prevalence of kidney stones and osteoporosis were similar in HS-PHPT and A-PHPT patients. Hypertension had the same rate in the two groups (66% vs. 60%, HS-PHPT vs. A-PHPT), and no difference in smoking habit was detected.

Regarding metabolic status, serum glucose and insulin levels, as well as HOMA-IR and occurrence of overt diabetes mellitus did not differ in the two groups. Moreover, though the prevalence of overt dyslipidemia was similarly detected in about one-third of PHPT patients, median serum LDL cholesterol level was slightly but significantly lower in HS-PHPT patients (Figure 1(e), panel).

**Figure 2:**
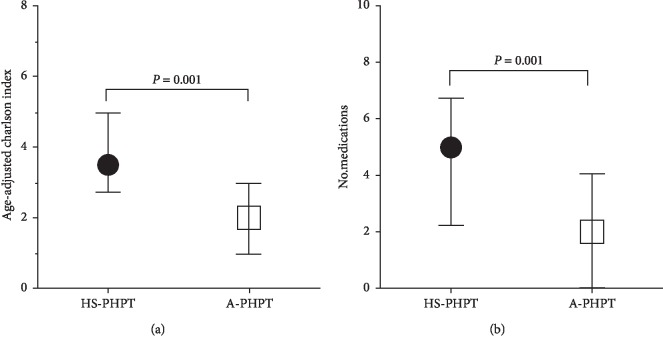
Differences in multimorbidity and polypharmacy between HS-PHPT and A-PHPT. Median values of age-adjusted Charlson index (a) and of the number of medications (b) differed between HS-PHPT and A-PHPT patients. Data are presented as median and range interquartile by box, and wisker plots represent minimum and maximum values. Statistical significance was determined by the Mann–Whitney test. HS-PHPT, hypercalcemic-related symptomatic PHPT (black circles); A-PHPT, asymptomatic PHPT (grey squares).

Finally, we calculated the age-adjusted Charlson comorbidity index, which is considered an indicator of patients' health status. The index was significantly higher in HS-PHPT patients (Table 1 and Figure 2(a), panel), suggesting that HS-PHPT patients had multiple concomitant comorbidities. Additionally, SH-PHPT patients were on chronic therapy with a greater number of medications than A-PHPT patients (Table 1 and Figure 2(b), panel).

Received operator characteristic curve (ROC curve) analysis confirmed the ability of the parameters age (area 0.665, *P*=0.028), eGFR (area 0.685, *P*=0.016), polypharmacy (area 0.728, *P*=0.001), and age-adjusted Charlson index (area 0.732, *P*=0.002) to be predictive of symptomatic hypercalcemia. The logistic regression analysis considering as dependent variable the occurrence of hypercalcemic-related symptoms and as independent variables age, serum calcium, PTH and ALP levels, eGFR, polypharmacy and age-adjusted Charlson index, showed that serum calcium levels were the strongest predictor of hypecalcemic-related symptoms in PHPT patients with an Odd Ratio (OR) of 2.73 (*P*=0.013), though polypharmacy and age-adjusted Charlson index also were predictive of hypercalcemic-related symptoms with ORs of 1.50 (*P*=0.05) and 2.46 (*P*=0.05), respectively.

## 4. Discussion

The consensus statements from the first PARAT workshop, a new European Society of Endocrinology program, aiming to identify unmet scientific and educational needs of parathyroid disorders, held in September 2018, highlighted that evidence concerning the natural history of PHPT and whether morbidity and long-term outcomes are related to hypercalcemia or plasma PTH concentrations, or both, is yet sparse [9]. Present data aimed to contribute to these concerns. PHPT patients attending with asymptomatic primary hyperparathyroidism (PHPT) are common occurrence in the outpatient endocrine setting, and clinicians may be no longer usual in recognizing hypercalcemia-related symptoms in PHPT patients, leading to underrate the prevalence. In the present Italian series of PHPT patients, consecutively enrolled in a single academic hospital equipped with emergency department, hypercalcemia-related symptoms occurred in 16% of cases, suggesting that in this setting, hypercalcemia-related symptoms are not unusual. Main symptoms were gastrointestinal, neurologic, and cognitive impairment, while heart failure and sepsis may occur. The clinical severity of the symptoms required hospitalization in two-thirds of cases. Nonetheless, short-term hypercalcemia-related mortality was low (1 out of 18 cases).

Investigating the clinical characteristics of the patients experiencing symptomatic hypercalcemia (HS-PHPT) compared with A-PHPT patients, HS-PHPT patients emerged as older and presented with a more severe hyperparathyroidism, in terms of higher serum calcium, PTH, and total ALP levels, suggesting that hypercalcemia-related symptoms are positively correlated with the parathyroid tumor activity. At variance, classical PHPT-related symptoms, namely kidney stone and osteoporosis, occurred with similar rates in HS-PHPT and A-PHPT patients, in line with recent reports, where end-organ PHPT manifestations (osteoporosis, nephrolithiasis, and hypercalciuria) developed before biochemical diagnosis or within 5 years in most patients, regardless of severity of hypercalcemia [10, 11].

Of note, kidney function was markedly reduced in HS-PHPT. This might be related to older ages and PHPT clinical severity, as hypercalciuria-induced nephrogenic diabetes insipidus often results in polyuria leading to volume depletion and a reduction in the glomerular filtration rate, which may lead to a further increase in calcium concentration [12]. Similarly, a retrospective study of 160 PHPT cases managed at a regional centre in the United Kingdom reported that higher peak calcium concentration was an independent predictor of acute kidney injury [13].

Therefore, patients experiencing symptomatic hypercalcemia were older, with a reduced kidney function, experiencing higher comorbidity score and treated with a higher number of drugs. In this analysis, we operationally define multiple chronic diseases, using the Charlson comorbidity index, intrinsically a measure of aggregate chronic disease burden, developed to predict survival and validated for diseases, like cancers and diabetes [4]. Multimorbidity and polypharmacy are associated with mortality, incident disability, hospitalization, and emergency department visits in frail and prefrail older adults [14–16]. Indeed, after adjustment for age and eGFR, logistic regression analysis showed that serum calcium levels are predictive of the risk to develop symptomatic hypercalcemia; however, polypharmacy and multimorbidity are factors contributing to the risk.

Therefore, data from the present series suggested that patients with severe PHPT should be carefully evaluated for concomitant multimorbidity and polypharmacy in order to estimate the risk of symptomatic hypercalcemia.

Treatment of symptomatic PHPT was surgical in more than a half of HS-PHPT patients: success rate was high, determining serum calcium normalization in more than 90% of cases. In patients contraindicated for or refusing parathyroidectomy, medical treatment with cinacalcet HCl was effective in controlling calcemia. We are tempted to suggest that patients with multimorbidity and polypharmacy at diagnosis of PHPT should be surgically treated, in agreement with recent advice [17], or, if contraindicated or refused, should be carefully followed-up for the development of symptomatic hypercalcemia. Cinacalcet HCl may be considered a therapeutic option to avoid symptomatic hypercalcemia.

## 5. Conclusions

PHPT patients experiencing hypercalcemia-related symptoms are not uncommon. Hypercalcemia-related symptoms are associated with severity of the parathyroid tumor function, multimorbidity, and polypharmacy. Clinicians should investigate all these factors in PHPT patients in order to estimate the risk of developing hypercalcemic-related symptoms and to consider parathyroid surgery.

## Figures and Tables

**Table 1:** Clinical and biochemical differences between PHPT patients experiencing hypercalcemic-related symptoms and asymptomatic PHPT patients.

	Reference range	HS-PHPT	A-PHPT	*P*
Patients, *n*		18	94	
Age, years		73.0 (81.5–91.5)	65.0 (55.5–74.0)	**0.026**
Gender, M/F		3/15	18/76	0.805
BMI, kg/m^2^		25.0 (21.0–28.6)	26.5 (23.2–29.7)	0.504
MEN1-related PHPT, %		0.0	8.5	1.000
*Calcium phosphate metabolism-related biochemical parameters*				
Serum calcium, mg/dl^*∗*^	8.4–10.4	12.5 (11.3–13.7)	10.6 (10.3–11.3)	**0.001**
Ionized calcium, mmol/L	1.18–1.30	1.52 (1.43–1.91)	1.39 (1.28–1.47)	**0.001**
PTH, pg/ml	10.0–65.0	422.0 (200.0–704.0)	130.0 (90.7–189.8)	**0.001**
25OHD, ng/ml	>30.0	14.0 (7.1–22.2)	20.1 (10.3–26.7)	0.126
Total ALP, U/L		104.0 (92.2–122.3)	79.5 (60.2–116.0)	**0.009**
Bone-specific ALP, *μ*g/L	6.0–26.0	33.8 (19.9–42.1)	14.8 (10.8–28.2)	**0.010**
eGFR, ml/min		71.0 (43.0–96.0)	90.5 (71.8–104.8)	**0.015**
eGFR < 45 ml/min, %		42.0	5.0	**0.050**
Serum phosphate, mg/dl	3.50–5.00	2.40 (2.08–2.71)	2.60 (2.20–2.90)	0.371
Urine calcium, mg/kg/24 h	<4.00	4.59 (2.85–6.98)	4.10 (2.80–5.76)	0.520
Urine phosphate, g/24 h	<1.00	0.49 (0.36–0.86)	0.73 (0.56–0.89)	**0.028**
*PHPT-related clinical features*				
Kidney stones, %		44.0	51.0	0.615
Osteoporosis, %		50.0	52.0	1.000
Hypertension, %		66.0	60.0	0.611
Smokers, %		8.3	13.5	1.000
*Metabolic status*				
Glucose, mg/dl	>100.0	91.0 (81.0–104.5)	90.0 (81.0–96.3)	0.921
Insulin, mU/L	5–25	11.0 (5.9–15.3)	7.3 (2.8–11.6)	0.146
HOMA-IR	<2.5	2.56 (1.09–3.53)	1.62 (0.52–2.85)	0.219
Overt diabetes, %		16.7	5.3	0.087
Total cholesterol, mg/dl	<200.0	188.5 (150.5–211.8)	195.5 (172.8–223.0)	0.130
HDL cholesterol, mg/dl	>40M, >50F	59.0 (46.2–73.7)	56.0 (47.2–67.5)	0.691
LDL cholesterol, mg/dl	<130.0	106.1 (75.9–117.9)	114.1 (94.8–139.9)	**0.033**
Triglycerides, mg/dl	<150.0	117.0 (92.2–135.5)	98.5 (74.5–123.3)	0.118
Overt dyslipidemia, %		27.0	36.0	0.593
*PHPT patients' health status*				
Age-adjusted Charlson comorbidity index		3.5 (2.7–5.0)	2.0 (1.0–3.0)	**0.001**
Chronic medications, *n*		5.0 (2.2–6.8)	2.0 (0.0–4.0)	**0.001**

HS-PHPT, hypercalcemic-related symptomatic PHPT patients; A-PHPT, asymptomatic PHPT patients; M/F, male/female; ^*∗*^albumin-corrected serum calcium; PTH, parathormone; ALP, alkaline phosphatase; eGFR, estimated glomerular filtration rate; and 25OHD, 25hydroxy-vitamin D.

## Data Availability

The datasets generated during and/or analysed during the current study are available from the corresponding author on reasonable request.
